# Predictors of malnutrition among older adults aged above 65 years in eastern Ethiopia: neglected public health concern

**DOI:** 10.1186/s12877-020-01911-2

**Published:** 2020-11-23

**Authors:** Abdu Oumer Abdu, Imam Dagne Yimamu, Ahmed Ale Kahsay

**Affiliations:** 1 Department of Public Health, Dire Dawa University, Dire Dawa, Ethiopia; 2 School of Medicine, Dire Dawa University, Dire Dawa, Ethiopia

**Keywords:** Malnutrition, Older adults, Mini nutritional assessment, Ethiopia

## Abstract

**Background:**

A nutritional problem, especially under nutrition is one of the common public health problems in older population causing greater mortality and economic loss in developing countries. However, evidences on the risk factors for increased nutritional risk among older population is not well stated in Ethiopia. This study aimed to assess the nutritional status and predictors of malnutrition among older adults (> = 65 years) in Eastern Ethiopia.

**Methods:**

A community-based analytical survey was conducted among randomly selected 592 older people aged above 65 years of age in Harari region. Subjects were selected using multistage sampling pretested Full Mini Nutritional Assessment (MNA) tool was used to classify as malnourished (MNA score < 17), at risk of malnutrition (MNA score of 17 to 23.5) and otherwise normal. Validated geriatric depression scale short form (15 items) was employed to screen for depression. Data were presented using statistical tables, frequency, percentage, and graphs. Ordinary logistic regression was employed to identify predictors of malnutrition and plum method was used to generate odds ratio. The level of statistical significance was declared at *P*-value less than 5%. Chi-square test, crude and adjusted odds ratio with 95% confidence was reported.

**Results:**

A total of 592 respondents (93.4%) were interviewed. About 306 (51.7%) and 93 (15.7%) were found to be at risk of malnutrition and malnourished respectively. The predicted log odds of being malnourished was higher among those from rural residents (AOR = 2.08: 1.25–3.45), not on working (AOR = 1.31: 95% CI: 0.87–1.95) and did not have health insurance (AOR = 1.58; 95% CI; 0.97–2.58). Those with chronic pain (AOR = 1.70; 95% CI: 1.15–2.51), previous hospitalization (AOR = 1.59: 95% CI: 1.27–2.38) and not able to cover their personal expense (AOR =1.61: 95% CI: 1.12–2.30) were predictors of malnutrition. The relationship between previous hospitalizations with malnutrition among older adults people is moderated significantly by the presence of chronic pain (β = 0.113, *p* = 0.015).

**Conclusions:**

Malnutrition among old age is a public health concern that needs attention. Economical vulnerability, residence, depression, presence of chronic disease, and hospitalization were important risk factors for malnutrition among old age.

**Supplementary Information:**

The online version contains supplementary material available at 10.1186/s12877-020-01911-2.

## Background

Owning to demographic transition and advances in medical technologies, the world population structure is changing. Worldwide as of 2015, there are 617.1 million older peoples above 65 years with almost 7.6% of the world Population [1]. In Africa, for example, the life expectancy has risen to about 65 years and accounts for 3.5% (40million) in 2015 [2]. While, in Ethiopia, 3.2% of the population were older than 65 years [3].

Global health issues for older age include nutritional problems, chronic non-communicable diseases like heart disease, stroke, and others. A nutritional problem, especially undernutrition is one of the common public health problems in the older adults causing greater mortality and economic loss facing the older population in developing countries [4]. Ethiopia is a country with emerging nutritional problems with its adverse health consequences [4, 5]. Early detection of malnourished and at risk for malnutrition will be valuable in targeting public health interventions among the older population.

Mini Nutritional Assessment (MNA) is an important validated tool used to assess older adults who are malnourished and at risk of malnutrition. It will be administered in MNA short form (MNA-SF) or the full-length screening tools (18 items) [6, 7] even if, both have been validated for use in malnutrition screening among old age population. It helps to identify malnutrition early before severe malnutrition with protein depletion happens. It has better performance in sensitivity and specify than Malnutrition Universal Screening Tools in the identification of malnourished ones [8].

Nowadays due to numerous social structural changes, this segment of the population is being with less care. Also only half of the older adults people have a private house while, about 10% live without a private home, in mosques and churches [9]. It is estimated that 15–50% of the older adults are affected by malnutrition in the community while in hospital settings 20–60% of older adults were affected by malnutrition [10].

In Ethiopia, almost a third of the older adults has one or more visual, mobility, weakness, or hearing difficulty which affects people’s access, and utilization of food. Moreover, some older adults have pension while the majority of the rural depends on their relative for support [9].

Globally, malnutrition is estimated to affect one in every six population; it is still increasing along with increased aging [7]. Especially, those with cognitive decline, hospitalized or institutionalized are at greater risk of malnutrition than community dwellers [7]. Similarly, malnutrition results in twice the risk of long term mortality, three times increased the risk of infection and longer period of hospitalization, increasing the health care costs. Malnutrition among older adults also increase the rate of hospital readmission by 30% which places huge financial burdens on government and individuals [11]. Despite this huge burden, malnutrition is still underreported due to lack of regular malnutrition screening [8].

Malnutrition among old age above 65 years has a devastating impact on morbidity, mortality, increasing the health care cost [8]. A follow-up study showed that those old age at risk of malnutrition and malnourished had 60 and 33.7% cumulative survival after 3 years. Also, the hazards of all-cause mortality were increased from 56 to 71% [12].

Despite malnutrition is being major public health problem among older adults, there are a limited studies in Ethiopia [12, 13] showing malnutrition is a common problem affecting from 9 to 22% in the northwest Ethiopia [14]. Along with limited studies, previous studies were unable to use an appropriate screening tools and unable to pinpoint major predictors of malnutrition among the older adults in Ethiopian setting for action to be taken. Thus, particular research was to generate reliable evidences on magnitude of malnutrition and its context-specific risk factors using reliable and validated tools for better geriatric health.

### Objective of the study

- ✓To assess the nutritional status and predictors of malnutrition among older adults (> = 65 years) residing in Harari region, Eastern Ethiopia.

## Methods

### Study area and period

This study was conducted in Harari region, Eastern Parts of Ethiopia. Harar is found about 526kms far from Addis Ababa, capital city of Ethiopia. According to the Ethiopian central statics authority’s 2007 report, Harari region has a total population of 183, 344 of whom 92,258 were men and 91,086 women and majority of its population lives in 99,321 or 54.17% of the population lives in urban (CSA 2007 report). Ethnic groups in the region include the Oromo (52.3%), Amhara (32.6%), Harari (7.1%) and others like Tigre and Guraghe etc. According to 2010 Harari Region population projection there are 250, 093 with 146, 913 living in urban and 122, 942 are male with total house hold of 64, 334.

According to Ethiopian demography, 2018 [15], in Ethiopia 2.91% of the population are Older adults above 65 years of age. Using this conversion factor the expected number of older adults was calculated and estimated. This Quantitative cross sectional study was conducted from March 01 to 30, 2019.

### Population and eligibility

All older adults aged > = 65 years of age in Harar region were the target population for this study to which the result is considered to be applied. While, those randomly selected people age greater than or equals to 65 years from the selected kebeles, were study population and included in the current survey. Those community dwellers aged above or equals to 65 years with or without their care givers residing in the selected kebles (lower administrative unit in Ethiopia) were included in the current survey. Those older adults who have no any caregiver and unable to communicate and give information were excluded from the study as we are unable to get reliable data from them. In addition, those who were not volunteers to take part in the survey were also excluded from the study. Study subjects with severe spinal curvature (kyphosis or scoliosis), both extremities amputation were not included in the study as this makes plausible measurement of height difficult for reliable body mass index measurement.

### Sample size determination

To determine the minimum sample size for the first objective, single proportion samples size formula with P as prevalence of malnutrition from the previous study (21.9) [14], 95% confidence level, “Z” critical value at 95% CI and marginal error of “d” 5% and became 263.

$$ n={\frac{\left( Z\alpha /2\right)}{}}^2\frac{P\left(1-P\right)}{d^2} $$

For factors associated with malnutrition the sample size is determined using OpenEpiSave software for cross sectional survey taking empirical statistics like odds ratio, proportion of exposed with malnutrition and power of 80%, with ratio of exposed to non-exposed as 1 and 5% level of significance. Taking the larger sample size calculated from objective two (286), design effect of 2, and 10% non-response rate, the final sample size was (572 + 0.1(572)) = 630. Thus, this study tried to interview and include 630 study subjects (older adults).

### Sampling procedures

Multi stage sampling was used to select eligible older adults from randomly selected Kebles from each Woreda. Then from each selected woredas using simple random sampling, we select two Kebles randomly from each woreda. Then the sample size was proportionally allocated for each respective selected woredas and then to the selected Kebles. The allocated number of older adults were interviewed from each randomly selected kebeles. Then, the data collector located the center of the kebeles and then randomly select a random direction using random spinning a pen. Then all HHs with older adults were interviewed in that selected direction until the sample size is achieved. However, when the required sample size is not achieved, another random direction were selected in the similar way and data collected similarly.

### Data collection methods

Data were collected using set of structured questionnaires including socio demographic situations, full mini nutritional Assessment (MNA-full) [7], geriatrics depression scale (GDS-SF), psycho social issues and others. The contextualized data collection tool is attached as supplementary file with this paper (English Version of Questionnaire). Data were collected by trained graduating health science students from house to house visit. Data collectors interviewed the elders and/or the care giver where communication become difficult. The interview was administered in respondent’s language they can.

The full MNA tool is worldwide validated too with 80% specificity and 90% sensitivity making it as the best, effective, affordable and quick malnutrition screening tool among older adults. It has also showing greater importance in identifying overt malnutrition and risk of malnutrition early, for effective public health interventions [16]. Thus the full MNA tool was contextualized, translated to Amharic and pretested before data collection. The full MNA score tool approved by Nestle Institute which contain 18 items were used with translation and it is published in [7].

The weight of the subjects were measured using calibrated electronic weighting scale to the nearest 0.1 kg. The height was measured using adult stadiometre for those who can stand. While for those who are unable to stand the Arm span from the sterna notch to tip of finger or knee height was to be used as proxy indicator for height of the subjects using specific formula for the specific sex, ethnic group. The BMI was calculated by dividing the weight in Kg by the height in m square and was expressed in kg/m^2^. However when the height measurement is not possible, calf circumference was used instead of BMI and the status was classified according to the nestle recommendations [7].

The mid upper arm circumference (MUAC) was measured using non-stretchable tape meter on the left arm at midpoint between the acromion process of clavicle and elbow joint. It was measured in arm extended and recorded in centimeter. Short twenty four hour dietary recall was used to assess the dietary intake pattern of the clients, as it reduce the recall bias secondary to memory lapse.

The geriatric depression score was used to assess the psychological condition off the older adults. A fifteen item depression scale assessment were used by direct interviewing the respondent. This tool has shown almost equal sensitivity in identifying depression level which have direct influence on malnutrition among older adults.

### Data quality assurance

Pair of trained graduating health students were employed to collect the data from study subjects as anthropometric measurement need curiosity and two individuals during measurement. Two day training appropriate interview techniques, anthropometric measurements like height and weight, practices were performed before actual data collection. After that constructive feedbacks were given for the data collectors by investigators and supervisor until they become clear of the checklist implementation. Anthropometric reliability assessment were done on 10 study subjects and inter and interobserver variation were calculated. Cranach’s Alpha measure of reliability used and kappa above 0.7 were considered acceptable and all within the acceptable range. All standard measuring procedures and instruments were strictly followed while data collection. During data entry in to EpiData the data quality was kept by making legal ranges, skipping patterns, appropriate coding and careful data entry. The intra observer and inter observers technical error of measurement were calculated after training of the data collectors and supervisors, to measure the reliability of the weight and height anthropometric measurements.

### Methods of data analysis

After checking for completeness ad inconsistencies, the collected data were entered in to EpiData Version 3.02 and Exported to SPSS version 20.0 for analysis. The data is presented in tables, graphs, percentages, frequencies, mean, medians and standard deviations. After measurement of weight and height, body mass index was calculated automatically. Similarly geriatric depression score was computed using the compute command. The outcome variable malnutrition status was categorized as those with malnutrition, at risk of malnutrition and normal nutritional status based on the overall sum score of each subject. Malnutrition was assessed using the full MNA score (out of 30) and calculated using the compute command in SPSS. Those who scored below 17 (malnourished), 17 to 23.5 (at risk of malnutrition) and otherwise normal [7]. At the same time the GDS was calculated using 15 items yes or no (1- yes for the presence of one of the depression symptoms. A GDS of 10 to 15 were considered as severe while, 5 to 9 as mild depression and no depression otherwise.

Then both bivariate and multivariable ordinal logistic regression were conducted using crude and adjusted odds Ratio at 95% confidence interval reported. The odds ratio corresponding to each variable were calculated using PLum command and by exporting the result to excel as needed. Those variables with *P* value less than 0.20 and important predictors in bivariate analysis were taken to multivariable ordinal logistic regression analysis. P value less than 0.05 at multivariable analysis were declared as statistically significant association.

### Variables

Nutritional status of older adults (ordinal scale) was the dependent variable whereas, demographic variables (age, sex, income, occupation, pension, education, and parental support), smoking, depression, appetite, chronic disease, oral health problems and physical exercise were independent variables.

### Ethical considerations

Ethical clearance was taken from Dire Dawa University Research and Technology Interchange directorate. Then, it was forwarded to Harari health bureau then to respective woredas and Kebles. Verbal informed consent were obtained from the respondents who are able to give consent otherwise it will be taken from caregivers or their parents. The data is used only for this research only and also in the future. Personal identification of clients like name, personal location and others were not be recorded. For those having severe malnutrition, nutritional counseling were done. In addition, counseling in order to have health care service in the nearest health facility were advised in advance for them and caregiver.

## Results

### Basic characteristics of participants

In this particular study, a total of 592 respondents were recorded with overall response rate of 93.4%. Regarding religion, a total of 290 (50.3%) and 244 (42.4%) were followers of Islam and orthodox Christianity respectively. Almost half, 292 (49.3%) were married followed by 212 (35.8) of their partner has died. Majority of them, 316 (54%) were illiterate while, 176(30.1% attended primary school. Majority of respondents, 508 (85.8%) and 522 (88.2%) were from rural area and live with their relatives respectively. With respect to occupation of respondents, 308(52%) and 209 (35.3%) reported that they do not have work and involved in private work. A total of 406 (69.2%) reported that they did not have any pension fee (Table 1). Regarding perceived health status of older adults, about 37, 47 and 16% of them perceived as good, average and poor respectively.

**Table 1:** Basic characteristics of elderly peoples in Harari Region, Eastern Ethiopia, 2019

Characteristics	Number	Percent
Sex (*n* = 592)		
Male	291	49.2
Female	301	50.8
Religion (*n* = 576)		
Muslim	290	50.3
Orthodox	244	42.4
Protestant	40	6.9
Catholic	2	0.3
Marital Status (*n* = 592)		
Married	292	49.3
Single	18	3.0
Divorced	70	11.8
Widowed	212	35.8
Educational Status (*n* = 585)		
Illiterate	316	54.0
Primary School	176	30.1
Grade 8–12	66	11.3
College And Above	27	4.6
Residence (*n* = 592)		
Urban	84	14.2
Rural	508	85.8
Live With (*n* = 592)		
Alone	70	11.8
With Others	522	88.2
Do You Work At The Moment Pension Fee (*n* = 587)		
Yes	271	46.2
No	316	53.8
Who Cover Your Expense (*n* = 585)		
Yes	258	43.6
No	327	55.2
Health Insurance (*n* = 586)		
Yes	94	16
No	492	84
Occupation (*n* = 590)		
Farmer	39	6.6
Private	209	35.3
Government	34	5.7
Not Have Work	308	52.0
Pension Fee (*n* = 587)		
Yes	168	28.6
No	406	69.2
Not Reached	13	2.2

Regarding lifestyle characteristics, a total of 210 (35.5%) had habit of khat chewing. While about 48 (8.1%) smoke cigarette. In addition, 349 (59%) and 330 (55.7%) had at least one chronic illness and insomnia respectively with 196 (33.1%) had history of hospitalization in the previous year. However, only 135 (22.8%) had habit of physical activity. More than half, 381(644%) reported to have some sort of dental problem or eating problem (Table 2).

**Table 2:** Factors associated with Malnutrition among elderly people in Harari region, Eastern Ethiopia

Factors	Nutritional status						χ2 (*p* value)
	Malnourished No (freq.)		At risk of malnutrition No (freq.)		Normal No (freq.)		
Sex							
Male	43	15%	147	51%	101	35%	1.16 (0.281)
Female	50	17%	159	53%	92	31%	
Live with whom							
Alone	8	11%	37	53%	25	36%	0.95 (0.329)
With others	85	16%	269	52%	168	32%	
Religion							
Muslim	69	24%	151	52%	70	24%	34.2 (0.000)
Christian	24	8%	155	51%	123	41%	
Marital status							
Married	24	8%	157	54%	111	38%	42.3 (0.000)
Divorced	13	15%	55	63%	20	23%	
Widowed	56	26%	94	44%	62	29%	
Educational status							
Illiterate	76	24%	153	48%	87	28%	32.16 (0.000)
Primary school	13	7%	104	59%	59	34%	
Grade8 & above	4	4%	49	49%	47	47%	
Work at the moment							
Yes	11	4%	144	53%	116	43%	53.4 (0.000)
No	80	25%	162	51%	74	23%	
Occupation							
Farmer	1	3%	19	49%	19	49%	47.60 (0.000)*
Private	11	5%	111	53%	87	42%	
Government	1	3%	20	59%	13	38%	
Not have work	79	26%	155	50%	74	24%	
Residence							
Urban	4	5%	35	43%	42	52%	18.41 (0.000)
Rural	87	17%	271	53%	150	30%	
Who covers your expense?							
Yes	13	5%	140	54%	105	41%	6.16 (0.013)*
No	77	24%	164	50%	86	26%	
Health insurance							
Yes	5	5%	53	56%	36	38%	34.6 (0.000)
No	85	17%	252	51%	155	32%	
Pension fee							
Yes	34	20%	85	51%	49	29%	3.3 (0.071)*
No	59	14%	221	52%	144	34%	
Chronic pain							
Yes	89	26%	179	51%	81	23%	67.1 (0.0001)
No	4	2%	127	52%	112	46%	
Insomnia							
Yes	71	22%	169	51%	90	27%	20.3 (0.0001)
No	22	8%	137	52%	103	39%	
Khat chewing							
Yes	20	10%	114	54%	76	36%	6.58 (0.01)
No	72	19%	192	50%	117	31%	
Smoking							
Yes	71	22%	169	51%	90	27%	4.26 (0.039)
No	22	8%	137	52%	103	39%	
Hospitalization in previous year							
Yes	60	31%	99	51%	37	19%	57.3(0.0001)
No	33	8%	207	52%	156	39%	
Chewing problem							
Yes	70	18%	197	52%	114	30%	6.7 (0.01)
No	23	11%	109	52%	79	37%	
Physical exercise							
Yes	15	11%	75	56%	45	33%	1.2 (0.296)
No	78	17%	231	51%	148	32%	
Depression							
Depression	75	40%	84	45%	28	15%	106.02 (0.0001)
No depression	18	4%	222	55%	165	41%	

In general, a total of 187 (31.6%) of respondents had screening positive for geriatric depression with 131 (22.1%) and 56 (9.5%) had moderate (GDS score of 5 to 9) and severe depression (GDS score of 10 to 15) respectively. While, more than half, 405 (68.4%) were negative (no depression) for Geriatric depression screening tool short version (Table 3).

**Table 3:** Detail report on the outcomes of GDS short form screening for elderly people in Harari Region, Eastern Ethiopia

GDS dimensions		Frequency	Percentage
Are You basically Satisfied with your life?	Yes	526	88.9
	No	66	11.1
Have you dropped many of your activities and interests?	Yes	197	33.3
	No	395	66.7
Do you feel that your life is empty?	Yes	74	12.5
	No	518	87.5
Do you often got bored?	Yes	118	19.9
	No	474	80.1
Are you in a good sprit most of time?	Yes	421	71.1
	No	171	28.9
Are you afraid that something bad is going to happen to you?	Yes	112	18.9
	No	480	81.1
Do you feel happy most of the time?	Yes	444	75.0
	No	148	25.0
Do you often feel hopeless?	Yes	100	16.9
	No	492	83.1
Do you prefer to stay at home rather than going out?	Yes	227	38.3
	No	365	61.7
Do you feel that you have more problem with memorythan most?	Yes	140	23.6
	No	452	76.4
Do you think it is wonderful to be alive now?	Yes	444	75.0
	No	148	25.0
Do you feel worthless the way you are now?	Yes	87	14.7
	No	505	85.3
Do you feel full of energy?	Yes	298	50.3
	No	294	49.7
Do you feel that your situation is hopeless?	Yes	81	13.7
	No	511	86.3
Do you think that most people are better off than you are?	Yes	173	29.2
	No	419	70.8

### Nutritional status of older adults people

**Fig. 1:**
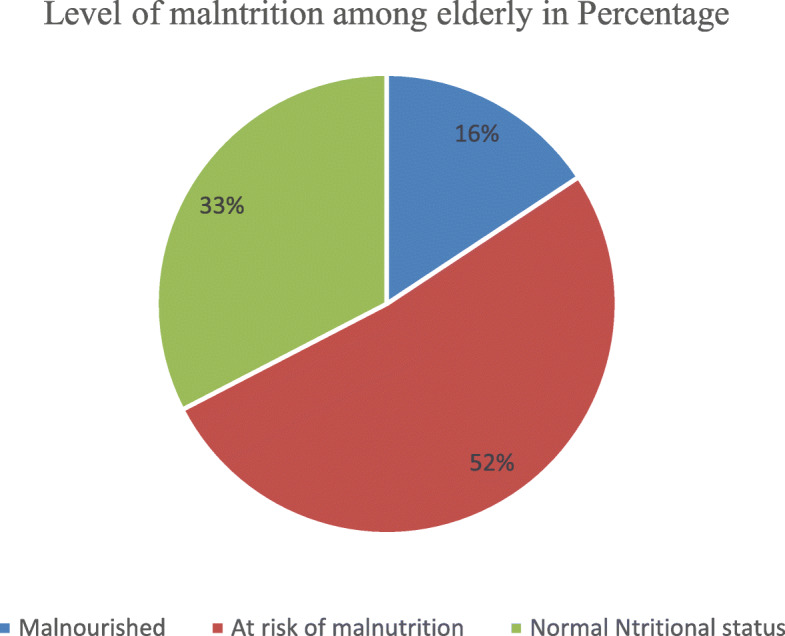
Nutritional status of elderly people in Harari Region, Eastern Ethiopia (*n* = 592)

In this study, majority 306 (51.7%) and 193 (32.1%) were found to be at risk of malnutrition (MNA score from 17 to 24) and not malnourished (MNA score greater than or equals to 24). While, 93 (15.7%) were malnourished (MNA score below 17) according to full MNA malnutrition screening tool (Fig. 1).

### Factors associated with malnutrition among older adults

Those respondents on pension, residing rural, with economic shortages, without health insurance, suffering from chronic pain, previous hospitalizations and positive for depression were predictors of malnutrition among older adults. Old ages from rural had higher log odds for being malnourished. Similarly, older age with history of chronic pain (COR = 170; *p* = 0.008) and hospitalization (COR =3.57; *p* value less than 0.0001) had four fold higher odds of being malnourished (Tables 2 and 4).

**Table 4:** Bivariate ordinal logistic regression output for prediction of malnutrition among elderly

Variables	β	Se f β	Exp (β) OR	*P*-value	95% CI (β)
Sex (female)	.172	.157	1.19	0.272	−0.14–0.48
Residence (Rural)	.997	.235	2.71	0.000	0.54–1.46
Education (higher)	−.565	.107	0.57	0.000	−0.78–−0.36
Living (with others)	.224	.243	1.25	0.357	−0.253–0.70
Marital status (separate)	.380	.088	1.46	0.000	0.21–0.55
Pension (yes)	.298	.176	1.35	0.091	−0.05–0.64
Work at the moment (No)	1.173	.168	3.23	0.000	0.85–1.50
Support (yes)	−.919	.165	0.40	0.000	−1.24–0.60
Health insurance (yes)	−.484	.216	0.62	0.025	−0.91–0.06
Chronic pain (Yes)	1.346	.171	3.84	0.000	1.01–1.68
Hospitalization (Yes)	1.272	.179	3.57	0.000	0.92–1.62
Insomnia (yes)	.700	.161	2.01	0.000	0.38–1.02
Physical activity (No)	.171	.187	1.19	0.359	−0.20–0.54
Depression (yes)	1.986	.200	7.29	0.000	1.59–2.38

The outcome variable, malnutrition is ordered in form of normal, at risk of malnutrition and malnourished. The OLS predict the probability of higher odds for malnutrition as compared to normal nutritional status by each predictor variables. The proportional odds assumption is partially full filled and other assumptions of OLS were met. The predicted regression coefficients are interpreted as predicted change in log odds of being malnourished as compared of normal per unit increase in predictor variables controlling for other variables. While, odds ratio is interpreted for categorical predictors.

The full model, including important predictors of malnutrition among Older adults, shows a significant improvement in model fitness under − 2 log likelihood for the intercept only and with the full model (P less than 0.0001). In addition, both the deviance (*p* = 0.08) and pearson chi-square test (*P* = 0.01) were not significant (above 5%) which showed better fitted OLS.

The predicted log odds of being malnourished (lower MNA score) was higher among those from rural residents (β = 0.73 (se = 0.21), *p* = 0.005). Older adults who are not on work and on pension (AOR = 1.31 95% CI: 0.87–1.95) and did not have health insurance (AOR = 1.58; 95% CI; 0.97–2.58) had 31 and 58% higher predicted odds of being malnourished than those who are on work respectively. In addition, those who have chronic pain history (AOR = 1.70; 95% CI: 1.15–2.51), previous hospitalization (AOR = 1.59: 95% CI: 1.27–2.38) and not able to cover their personal expense (AOR =1.61: 95% CI: 1.12–2.30) were 70, 60 and 61% significantly higher log odds of being malnourished (lower MNA score than higher) respectively. The relationship between previous hospitalizations with malnutrition among Older adults is moderated significantly by the presence of chronic pain (β = 0.113, *p* = 0.015) (Table 5).

**Table 5:** Parameters for predictors of malnutrition level as compared to normal and at risk of malnutrition (multivariable OLS model with stepwise backward regression method)

Predictors	β	Se f β	Exp (β) OR	*P* value	95% CI of Exp (β)
Pension fee user (yes)	*0.268*	*0.205*	*1.31*	*0.192*	*0.87–1.95*
Residence (rural)	*0.731*	*0.259*	*2.08*	*0.005*	*1.25–3.45*
Cover personal expense (no)	*0.474*	*0.185*	*1.61*	*0.010*	*1.12–2.30*
Have health Insurance (no)	*0.458*	*0.250*	*1.58*	*0.067*	*0.97–2.58*
Chronic pain (yes)	*0.532*	*0.199*	*1.70*	*0.008*	*1.15–2.51*
Hospitalization in the last year (yes)	*0.464*	*0.205*	*1.59*	*0.023*	*1.27–2.38*
Depression (yes)	*0.233*	*0.028*	*1.26*	*0.001*	*1.20–1.34*

## Discussion

This study was aimed to assess the magnitude of malnutrition risk and its predictors among the older adults in the eastern part of Ethiopia. The finding revealed that, 32.1% (28.2–35.8%) and 15.7% (12.8–18.6%) were at risk of malnutrition and malnourished based on validated MNA screening tool respectively. This is indicative of high risks of malnutrition and all its bad consequences in the overall health of individuals (12). Similarly, evidences from other regions of Ethiopia showed that, 28.3% [17], 22% [18], 21.9% [14], 17.6% (95% CI: 15.0–20.2) [19], 17.1% [20], 12.5% in Sirilanka [21] and 24.8% in Nepal [22] were malnourished.

This burden of malnutrition can still be above this, due to potentially higher incidence of risk factors for malnutrition. It might be expected that the burden will be higher among institutionalized elder people frailty in institutionalized persons (β: 0.22; *P* = 0.036) [23]. However, the current study incorporates both urban (4.9%) and rural area (17.1%) which makes representative. In addition, the use of BMI as tool to screen for malnutrition instead of MNA tool, may make a difference even if, it has high predictive power for malnutrition [17].

Rural residents were found to be at increased risk of being malnourished as compared to urban residents (AOR = 2.08; 95% CI: 1.25–3.45). This is attributed to differences in socioeconomic status, dietary habit and other confounding factors make the rural area at risk of malnutrition. It may also be related to lower access to health care, sanitation facility and educational status which makes disease, and food intake shortage a major problem [24, 25].

Economic dependences characterized by being pension user, not able to cover personal expenses and those without any health insurance were vulnerable to a higher probability of being malnourished. These economic instabilities decrease food access and dietary diversity ultimately resulting in malnutrition among the older adults. One study showed that less diversified diet intake increases risk of malnutrition among the older population [19]. This indicates, the need for strategies and interventions targeting the basic and underlying context-specific causes of malnutrition in addition to addressing immediate causes [26]. Other study, also showed that economic inaccessibility were an important risk factors for malnutrition burden [27]. Furthermore, evidence showed that presence of sustainable income is positively related to self-rated health status among old age (OR = 1.8) [28].

Depression is found to increase the nutritional risk among elders (AOR = 1.26: 95% CI: *1.20–1.34)*. One study indicated that, depression symptom significantly increase nutritional risk ((MNA score 22.86 vs. 24.96, *p* < 0.001) [29]. As depression is not a normal part of aging, rather common mood disorder that significantly affect the dietary intake and physiology which potentially affects the nutritional status. These may be related to increased nutrition risk due to hospitalization (*p*-value less than 0.0001) and not involved actively in work (p-value less than 0.0001) which makes them less active and at risk for depressive disorder. There should be screening and care for depression among older adults in health facilities. In addition, promoting active living style and reducing sedentary lifestyle could play an important role in reducing malnutrition and its adverse consequences [30–32].

Furthermore, the presence of chronic illness and hospitalization with multiple drug use are important predictors of malnutrition. A review of evidence showed that multiple drug intake negatively affects the nutritional status of the older adults (β: − 0.62; *P* = 0.001). Also, those elders in institutions other than home setting have increased risk of being malnourished [23, 31]. Thus, interventions developing positive lifestyles like physical activity and good dietary habit for reduced chronic diseases risk should get due attention. The inclusion older adults as target for nutrition and health interventions for better health status should be one target.

However, other confounding situations and variables may interact together to the deterioration of their nutritional status among institutionalized elders [23]. More specifically, the presence of chronic illness, poor caring practice and psychological status may affect food intake and nutritional status. In addition, polypharmacy may have the potential to decrease food intake and other side effects which might increases malnutrition risk [33].

The findings of this study employed validated tool with an overall accuracy of 91% (sensitivity and specificity of 87.9 and 89.6% for the established cutoff points) [34]. It also employed valid and reliable anthropometric measurements for better outputs. However, the cross-sectional nature of the study, some slight age-related disorders (curvatures) and measurement may create some errors. The result should be viewed in the light of the above strength and weakness of the study. But, the study pinpoints an important health problem of the neglected society in Ethiopia for programs to design and implement effective interventions addressing the older adults.

## Conclusions

Malnutrition among old age is a public health concern that needs attention. Economic vulnerability, residence, depression, presence of chronic disease, and hospitalization were important risk factors for malnutrition among old age. The health system needs to give attention to geriatric health and implement screening, health education, and follow-up of older adults’ nutritional status.

## Supplementary Information

**Additional file 1.**

## Data Availability

All data generated or analyzed during this study are included in this published article.

## Abbreviations

CI: Confidence Interval
EMR: Electronic Medical record
OLR: Ordinal Logistic Regression
OR: Odds Ratio
