# Supplementary material for: Predictors of malnutrition among older adults aged above 65 years in eastern Ethiopia: neglected public health concern
Source: BMC Geriatr. 2020 Nov 23;20:497. doi: 10.1186/s12877-020-01911-2 (PMC7684913; doi:10.1186/s12877-020-01911-2)
Supplement: Supplementary file 1 — Additional file 1. [file 12877_2020_1911_MOESM1_ESM.docx]

# English Version of Questionnaire

|  | Part I Socio demographic Identification | | | |  |
| --- | --- | --- | --- | --- | --- |
| S. No | Variables | Options | | | Remark |
| 101 | Identification Code |  | | |  |
| 102 | Sex | 1. Male 2. Female | | |  |
| 103 | Age | __________ years | | |  |
| 104 | Living Condition | 1. Alone 2. With Others | | |  |
| 105 | Religion | 1. Muslim 2. Orthodox 3. Protestant 4. Catholic 5. Others | | |  |
| 106 | Marital Status | 1. Married 2. Single 3. Divorced 4. Widowed 5. Others | | |  |
| 107 | Educational status | 1. Illiterate 2. Primary school 3. Grade 8-12 4. College and above | | |  |
| 108 | Currently Working | 1. Yes 2. No | | |  |
| 109 | Residence | 1. Rural 2. Urban | | |  |
| 110 | Monthly Income | ______________ Birr | | |  |
| 111 | Financial Dependency from other | 1. Yes 2. No | | |  |
| 112 | Do you have Health Insurance? | 1. Yes 2. No | | |  |
| 113 | Are you Pension user? | 1. Yes 2. No | | |  |
| 114 | Occupation | 1. Farmer 2. Self 3. Government 4. Not working | | |  |
|  | Do you chew khat | 1, yes ------- times/per week  2, No | | |  |
| 115 | How do you rate your current Health status? | 1. Good 2. Average 3. Poor | | |  |
|  | **Part II Health Condition of Respondents** | | | |  |
| 201 | Chronic Pain | 1. Yes 2. No | | |  |
| 202 | Insomnia | 1. Yes 2. No | | |  |
| 203 | Hospitalization During last Year | 1. Yes 2. No | | |  |
| 204 | No of chronic Disease | ______ | | |  |
| 205 | Number of drugs currently taking | ______ | | |  |
| 206 | Oral health status | | | |  |
|  | Chewing problem (partial or Complete) | | 1. Yes 2. No | |  |
|  | Edentulous (partial or Complete) | | 1. Yes 2. No | |  |
|  | Denture (partial or Complete) loss | | 1. Yes 2. No | |  |
| 207 | Physical Exercise | | 1. No 2. Occasionally 3. Daily (>=30 min) | |  |
| 301 | **Part III: GDS Questions** | | Yes | No |  |
| 301.1 | Are You basically Satisfied with your life? | |  |  |  |
| 301.2 | Have you dropped many of your activities and interests? | |  |  |  |
| 301.3 | Do you feel that your life is empty? | |  |  |  |
| 301.4 | Do you often got bored? | |  |  |  |
| 301.5 | Are you in a good sprit most of time? | |  |  |  |
| 301.6 | Are you afraid that something bad is going to happen to you? | |  |  |  |
| 301.7 | Do you feel happy most of the time? | |  |  |  |
| 301.8 | Do you often fee hopeless? | |  |  |  |
| 301.9 | Do you prefer to stay at home rather than going out and doing things? | |  |  |  |
| 301.1 | Do you feel that you have more problem with memory than most? | |  |  |  |
| 301.11 | Do you think it is wonderful to be alive now? | |  |  |  |
| 301.12 | Do you feel worthless the way you are now? | |  |  |  |
| 301.13 | Do you feel full of energy? | |  |  |  |
| 301.14 | Do you feel that your situation is hoppless? | |  |  |  |
| 301.15 | Do you think that most people are better off than you are? | |  | |  |

**Part IV: MNA Assessment Tool**

Complete the screen by filling in the boxes with the appropriate numbers.

|  | Date |  |  |
| --- | --- | --- | --- |
|  | Weight in kg | kg |  |
|  | Height in cm | cm |  |
|  | MUAC in cm | cm |  |
| A. | Has food intake declined over the past 3 months due to loss of appetite, digestive problems, chewing or  Swallowing difficulties? | 0 = severe decrease in food intake  1 = moderate decrease in food intake  2 = no decrease in food intake |  |
| B. | Weight loss during the last 3 months | 0 = weight loss greater than 3 kg (6.6 lbs)  1 = does not know  2 = weight loss between 1 and 3 kg (2.2 and 6.6 lbs)  3 = no weight loss |  |
| C. | Mobility | 0 = bed or chair bound  1 = able to get out of bed / chair but does not go out  2 = goes out |  |
| D. | Has suffered psychological stress or acute disease in the past 3 months? | 1 = Yes  2 = No |  |
| E. | Neuropsychological problems | 0 = severe dementia or depression  1 = mild dementia  2 = no psychological problems |  |
| F1. | Body Mass Index (BMI) (weight in kg) / (height in m) 2 | 0 = BMI less than 19  1 = BMI 19 to less than 21  2 = BMI 21 to less than 23  3 = BMI 23 or greater | Do not answer question F2 if question F1 is already completed. |
| F2. | Calf circumference (CC) in cm | 0 = CC less than 31  3 = CC 31 or greater | If BMI is not available, replace question F1 with question F2. |
| G. | Lives independently (Not in nursing home or Hospitals) | 1 = Yes  0 = No |  |
| H. | Take More than three Prescription Drugs per day? | 0 =Yes  1 = No |  |
| I | Pressure sores and Skin Ulcers? | 0 =Yes  1 = No | Clinical exam or Hx will be elicited |
| J. | How many Full meals does the client eat daily? | 0 = 1 meal  1 = 2 meals  2 = 3 meals |  |
| K. | Selected Consumption patterns for protein intake | At least one serving of dairy products (milks, cheese, Yoghurt) per day | Yes No |
|  |  | Two or more servings of legumes or eggs per week | Yes No |
|  |  | Meat, Fish and poultry every day | Yes No |
|  |  | 0 = If 0 or 1 yes  0.5 = If 2 yes  1 = If 3 yes |  |
| L. | Consumes two or more servings of fruits and vegetables | 0 = No  1 = Yes |  |
| M. | How much fluid (water, juice, coffee, milk…) is consumed per day? | 1. = less than 3 cups    1. = 3 to 5 cups   1.0 = more than 5 cups |  |
| N. | Mode of feeding? | 1. = Unable to eat without assistance 2. = self feed with assistance 3. = self feed without any problem |  |
| O. | Self view of Nutritional status | 0 = View self as being malnourished   1. = Is uncertain nutritional status 2. = view self as having no Nutritional problem |  |
| P | In comparison with other older people of the same age how do you consider your health status? | 1. = not as good   0.5 = does not know  1.0 = as good  2.0 = better |  |
| Q | Mid upper Arm circumference (MAC) in Cm | 1. = MUAC less than 21 cm   0.5 = MUAC 21 to 22 cm  1.0 = MUAC greater than 22 cm |  |
| R. | Calf circumference | 1. = CC less than 31 cm 2. = CC 31 cm or greater |  |
